# It’s All Critical: Acting Teachers’ Beliefs About Theater Classes

**DOI:** 10.3389/fpsyg.2020.00775

**Published:** 2020-05-19

**Authors:** Thalia R. Goldstein, DaSean L. Young, Brittany N. Thompson

**Affiliations:** Department of Psychology, George Mason University, Fairfax, VA, United States

**Keywords:** acting, theatre education, social skills, cognitive skills, teachers, arts education

## Abstract

Acting classes and theater education have long been framed as activities during which children can learn skills that transfer outside the acting classroom. A growing empirical literature provides evidence for acting classes’ efficacy in teaching vocabulary, narrative, empathy, theory of mind, and emotional control. Yet these studies have not been based in what is actually happening in the acting classroom, nor on what acting teachers report as their pedagogical strategies. Instead, previous work has been unsystematic and fragmented in its measured transfer outcomes, and absent mechanistic explanation. Expanding research on this topic requires more grounding in teachers’ beliefs about the acting classes they teach, as well as observation of the classes themselves. As a first step, we surveyed 173 acting teachers online, asking them about the activities within acting classes they believed caused change in their students, as well as which outcomes they believed were changed as a result of acting classes. Teachers taught across educational levels (elementary to professional) and had a variety of training in teaching acting. Overall, teachers rated almost every activity within classes as important for and causing impact on students, and almost every outcome as being positively influenced as a result of acting class. When forced to rank-order outcomes, teachers focused on collaboration, communication, creativity, confidence, and empathy as most likely to change. Teachers rated the importance of class activities and outcomes differently depending on what level they taught. This study shows the difficulty of surveying highly motivated teachers, given the globally high rankings, but also proposes candidate psychological skills likely to change as a result of acting classes and the mechanistic behaviors that may cause change.

## Introduction

Questions of whether and how learning within arts classrooms transfers to cognitive, social, and emotional abilities outside of those classrooms are fundamental to the study of arts education. In fact, arguments about the importance of arts education tend to be founded on their utility for other academic areas (Winner et al., 2013). In theater specifically, both theory and empirical study provide growing evidence of its positive effects in other domains. However, evidence is mixed across studies, and research methods vary in their ability to determine causality. In addition, theater practitioners and educators sometimes criticize research for being disconnected from the actual practices that occur in classrooms and for reducing the complex activity of theater to just a few measured variables (Omasta and Snyder-Young, 2014). Most studies are not based on a systematic, thorough investigation of which activities and behaviors are actually occurring in acting classrooms. One way to bridge this gap between researchers and practitioners, and to address critiques of prior work, is to directly survey stakeholders such as teachers about their perspectives on what occurs in classrooms that may transfer to general skills. Researchers can then use these results to structure future research studies. By asking theater teachers to report on what activities they use in their classrooms, and if they think those activities are important for transfer, as we do in the present study, researchers interested in acting can create a real-world, field-based starting point for understanding acting activities and their possible outcomes.

Previous research does provide evidence that overall learning within theater classrooms transfers to gains in other domains, particularly for verbal and social outcomes. A previous meta-analysis on the effects of theater training found that the only area with clear, causal, positive effects was verbal comprehension (Podlozny, 2000; Winner and Hetland, 2000). More recent research has found evidence of relationships between participating in theater and advancement of social–emotional outcomes such as theory of mind and empathy (Goldstein and Winner, 2012), emotional control (Goldstein and Lerner, 2017), emotion regulation (Larson and Brown, 2007; Goldstein et al., 2013), communication (Hui and Lau, 2006), narrative abilities (Nicolopoulou et al., 2015), and creativity (Sowden et al., 2015).

However, this extant research examines outcomes of theater classes without necessarily considering mechanisms of change. That is, previous work was not conducted based on specific behaviors and activities within an artistic practice or lesson but was, rather, holistically focused on “theater.” Studies that show effects of theater rarely describe classroom experiences that may have contributed to those effects (Kardash and Wright, 1987; Podlozny, 2000; Fleming et al., 2004; Mages, 2006; Goldstein and Winner, 2012). Nevertheless, theories of how specific acting activities are connected to outcomes proliferate. Mages (2006) hypothesized 13 separate drama activities which could result in changes in narrative and verbal outcomes (e.g., rehearsal and explanation of complex language, use of imagery), but did not come to any strong conclusions. Other work has highlighted physical movement, verbal interaction, and group work (Cawthon and Dawson, 2009, 2011) as well as motivation and explicit discussions of characters’ mental states (Goldstein and Winner, 2012) as possible mechanisms of change for student outcomes. Together, this previous theoretical and empirical work is missing a direct investigation of which psychological outcomes each possible mechanism may change.

To build a real theory of what acting may do for non-acting outcomes, researchers must consider the full landscape of a theater classroom and the goals teachers set within an acting class. This requires deeply analyzing the art form itself from a psychological perspective—to understand what is happening within it and therefore what students gain from participation. While such a study has been undertaken in the visual arts (Hetland et al., 2007), in music (Elliott et al., 2019), and in musical theater for children with autism spectrum disorders (Goldstein et al., 2019), to our knowledge, no such large-scale study has looked at theater. One study of a singular acting exercise focused on breaking down how a single exercise (“the 8 steps”) was linked to various cognitive and neuronal processes, in order to increase actors’ metacognitions about training (Lippi et al., 2016). Authors found that discussing acting concepts in psychological terms such as attention and neuronal mirror helped actors think about what their training was actually doing. A full-scale analysis of acting classes could illuminate possible psychological and behavioral mechanisms of change and help researchers better design studies to investigate how theater training transfers to other areas of development.

Therefore, in the current study, we focus on what theater teachers believe is happening in the classroom that leads to outcomes outside of the classroom, and what those outcomes could be. We look at all levels of theater teaching, from K–12 school-based programs to community, college, and professional programs. We take this research as a first step: before future studies can fully understand transfer, we must identify specific activities within these programs that might affect domains where transfer is expected. Similarly, rather than casting about for multiple domain general outcomes, foundational research must specify which social, cognitive, emotional, and behavioral outcomes may change directly as a result of theater training. Teachers are an ideal population with which to take this first step—they are often trained in theories of drama education and its effects, they develop and follow acting curricula without necessarily having transfer effects in mind (unlike researchers), and they have practical experience with children and adults who participate in acting classes, watching them learn and grow while teaching.

Although research considering stakeholder perspectives in theater is limited, one previous study surveyed a nationally representative sample of theater teachers (and school administrators), focused on availability of theater programs, and included a few questions on outcomes. This work identified confidence, creativity, collaboration, communication, and interpersonal skills as stakeholder-endorsed outcomes of theater programs (Omasta, 2012). Stakeholders clearly felt that theater played an important role in developing these qualities, but there were no questions about *how* theater might develop these skills. In one other small-scale study, teachers, teaching aides, and administrators of a musical theater program for children with autism spectrum disorder were surveyed about which activities within their program they felt affected change in their students and which changes they thought were happening as a result of their theater program. Mechanisms identified within the classroom were imitation, relaxation, small group work, and warm-ups. Teachers believed these activities helped change students’ imitation, motor, language, emotion recognition, and turn taking skills (Goldstein et al., 2019).

In the present study, we ask about both outcomes and mechanisms, with teachers rating the importance and the prevalence of each within their theater classrooms. We conceptualize outcomes from acting classes as social, emotional, cognitive, behavioral, and academic changes that occur as a direct result of participating in theater classes, such as skills described above as transferring from theater to other domains (e.g., verbal comprehension, social skills, creativity). We conceptualize mechanisms as the teaching techniques and classroom activities used by theater teachers that may facilitate change in students. Importantly, after rating the importance and prevalence of the listed outcomes and mechanisms (see Supplementary Appendix A), we asked teachers specifically which mechanisms are most responsible for change in the listed outcomes. In this way, we focus on classroom activities, transfer outcomes, and the connections between the two, both separately and together.

Because we are surveying teachers (rather than looking directly at classrooms or student outcomes), we also consider factors that might influence their answers, such as training and education, types of classes taught, and years of teaching experience. Critical to looking at the perspectives of teachers is also keeping in mind that teachers, parents, and program evaluators have a vested interest in the success of their programs (Eisner, 1997) and therefore may perceive many outcomes to be positively influenced by theater. Theater education is constantly under budgetary and educational threat. Therefore, teachers may be defensive about the activities within acting classes and the importance of their programs (Holcomb, 2007), and also may act as arts advocates who support and defend the impact arts can have in learning environments (Hayford and Kattwinkel, 2018; Robinson and Aronica, 2018).

## Materials and Methods

### Participants

Participants were recruited through a snowball method in a variety of ways, given the specific qualifications needed for participation (i.e., individuals with experience teaching acting). The authors posted survey links multiple times on social media sites (e.g., Facebook, Twitter), personally emailed contacts in theater departments and schools and asked those contacts to distribute the survey, and discussed the survey at conferences such as the American Psychological Association Annual Convention, asking audience members to take the survey or send around the link if they knew theater teachers.

As is common with online surveys, many participants did not complete the entire survey, and therefore, participant numbers are uneven section by section. A total of 375 participants completed the consent form, and approximately 174 participants completed the demographic information. All demographic information was used in describing the sample. In order to create the analytic sample, we included any participants who had answered the individual question of interest, allowing for the number of participants to vary between questions. The difference of the *N*s between questions of the same section did not exceed more than 3. At most, 216 participants answered questions about mechanism usage, 185 participants answered questions about mechanism importance, and 138 participants answered questions related to mechanism causality. For outcomes, 178 participants answered questions about theater’s effect on outcomes, and 148 participants ranked outcomes. For later inferential analyses, we then applied a filter to exclude participants who did not give usable information about their time split between age groups (e.g., did not respond, percentages did not equal to 100, or percentages exceeded 100), making the analytical sample, at most, 137 participants.

#### Participant Demographics

Of those who completed demographics, 32.9% identified as male, 66.5% as female, and 0.6% preferred not to answer. Participant mean age was 45 years old, with an SD of 12.6 years. Participants self-identified race (and could chose multiple categorizations): 91% self-identified as White; 2% Black; 5% Hispanic; 0.5% each Asian, Native American, and Native Hawaiian; and 0.5% declined to report race. Most of our sample was American, with 93% teaching in the United States and just single respondents working in other countries such as Italy, Ireland, Australia, Austria, China, and Netherlands.

#### Acting Training and Teaching

Participants were asked a variety of questions to ensure they had the requisite experience teaching theater. They were also asked whether they had earned a degree in theater and to describe the level and type of theater they taught. Allowed to choose multiple options, 30% had a degree in acting or performance, 12% in directing, 14% in theater education, and 17% in another type of education, including costume design, drama therapy, playwriting, dramaturgy, management, communication, music, psychology, and theater history. For level of education, 18% had an AA, BA, BS, or BFA; 66% an MA, MS, or MFA; and 16% a PhD, JD, MBA, or MD. Teachers taught acting classes at a variety of overlapping levels: 27% taught in elementary schools, 33% in middle schools, 52% in high schools, 49% in college or university settings, and 12% in community theater classes. In addition, 22% of participants had taught professional classes for children, and 12% had taught in professional conservatories for adults. Taken together, we were confident that all respondents had enough experience teaching acting classes to be able to answer questions about theater classes and possible student outcomes that result from those classes.

Participants were then asked to self-describe their time split among the various teaching level options described above. This was to investigate whether our sample spent time in early education, adolescent, or professional environments, as these environments have different demands for curriculum and engagement. The majority of participants spent the majority of their time teaching at the high school or college level. The mean percentage of time spent teaching youth (elementary and middle school) was 16.48% (*SD* = 31.11%); the mean percentage of time teaching adolescents (high school and college level) was 70.07% (*SD* = 39.79%); and the mean percentage of time teaching in professional schools (child or adult) was 10.96% (*SD* = 27.43%).

### Procedure

The link to the survey was anonymous, meaning it was the same link for all participants, and authors could not track individual responses. The link was directly to a Qualtrics (online survey platform) survey. The landing page was an online consent form describing the study and asking for informed consent, which was given by clicking “next” to proceed with the survey. Teachers were instructed as follows: “This research is being conducted to examine the cognitive, social and emotional skills being taught in theater classes for children and adolescents. If you agree to participate, you will be asked to complete the following survey which involves questions about the habits of mind being taught in theater classrooms, and the techniques used to teach such habits of mind.” Therefore, teachers were aware of the goal of the study. Participants completed the full survey, in order, beginning with rating classroom strategies, then outcomes, then linking the two, and finishing with demographics. IRB approval was given by George Mason University.

### Survey

The survey was created by the authors to assess theater teachers’ perceptions of different mechanistic activities and outcomes they believed to occur as a result of engaging in theater classes. Questions assessed perceptions in several ways to account for teacher bias toward the arts. Authors modeled part of the survey from previously published research and theory on acting classes (e.g., Goldstein et al., 2019) and also discussed and showed drafts of the survey to professors of theater education and high school–level acting teachers. Possibilities for the activities within acting classes were drawn from a wide variety of acting textbooks, including Spolin and Sills (1999) *Games for Theatre*, Stanislavski (1936) *An Actor Prepares*, Hull’s description of Lee Strasberg’s method (Hull, 1985), and texts on personal acting strategies and theories from Hagen (1973) and Meisner (1987). Outcomes were taken from the Core Arts Standards, as well as areas where researchers had previously published work, including meta-analyses. The final survey contained questions about 28 possible outcomes of theater education and 27 mechanistic activities within acting classrooms which may lead to outcomes. Types of mechanistic activities included various types of games (e.g., perspective taking, non-verbal, long- and short-form improvisation), types of modeling activities (e.g., by teacher, by peers, by video), physical activities (e.g., warm-up, calisthenics, meditations), rehearsal and performance activities (e.g., memorization activities, “speed through” lines, performance for the public, performance for classmates), and characterization activities (e.g., discussing characterization, sense memory, script analysis). Types of outcomes included social (e.g., eye contact, communication skills, empathy, turn taking), self-related (e.g., self-understanding, self-esteem, confidence, self-control, emotion regulation), physical skills (e.g., physical control, motor skills), and cognitive skills (e.g., memory, academic skills, focus, paying attention, language skills).

Teachers were first asked to rate mechanisms twice. They rated how often (from 1, not at all, to 7, multiple times per class) they used a mechanism and then rated its importance to the classroom (from 1, do not us, to 4, core to class). Then teachers rated outcomes twice. First by how much they believed each was positively affected by theater classes (from 1, not at all, to 7, a great deal) and then by a rank-order of the 28 outcomes from 1, most likely to change, to 28, least likely to change as a result of acting classes. Finally, teachers were asked about the explicit connection between mechanisms and outcomes by rating each mechanism’s contribution to creating change in outcomes (from 1, does not cause change, to 4, is the most critical aspect of acting classes causing change). Finally, participants were given an open-ended question, “Which kinds of activities are causing which kinds of changes?”, before completing demographic questions. Each outcome and each mechanism were presented in a random order by participant within each block of questions. The entire survey can be found in Supplementary Appendix A.

## Results

### Mechanistic Activities

See Table 1 for means, standard deviations, skewness, and kurtosis for all mechanism-related variables. Several of the mechanism variables were found to be non-normal (either skewness or kurtosis greater than 2; George and Mallery, 2009). Of the three ways (use, importance, and impact) participants were asked about mechanisms, issues of non-normality mostly occurred in response to being asked the importance of each of the mechanisms. Script/Character Analysis (skewness = -2.25, kurtosis = 5.27), Reflection (skewness = -2.03, kurtosis = 4.13), Exploring/Discussing Characterization (skewness = -2.18, kurtosis = 5.76), Performance for the Class (skewness = -2.32, kurtosis = 5.76), Social Interaction Games (skewness = -1.93, kurtosis = 4.08), and Rehearsing Work for Performance (skewness = -1.92, kurtosis = 3.75) were all negatively skewed and leptokurtic. The means for these variables were shifted to the upper half of the original scale, and data did not vary from around those shifted means. Put more simply, an overwhelming majority of teachers reported all of these mechanisms being very important to their classes. Practically all teachers endorsed many different activities at a very high rate.

**TABLE 1:** Descriptive statistics for mechanism variables (in alphabetical order).

		Often				Causal				Core		
Variables	Mean	SD	Skewness	Kurtosis	Mean	SD	Skewness	Kurtosis	Mean	SD	Skewness	Kurtosis
Body work	4.44	1.23	–0.12	0.04	2.62	0.71	0.31	–0.46	3.37	0.73	–1.30	2.01^c^
Define the language of acting, or define the language of a play/performance	4.64	1.25	–0.13	0.001	2.31	0.81	–0.04	–0.62	3.51	0.68	–1.36	1.80
Exploring/discussing characterization	4.79^a^	1.04	–0.03	0.65	2.91	0.70	–0.27	0.02	3.71	0.55	−2.18^b^	5.76^c^
Games and activities involving taking the perspective of other people	4.39	1.22	–0.15	0.24	3.04^a^	0.75	–0.17	–0.89	3.37	0.73	–1.12	1.19
Games and activities that involve non-verbal skills	4.10	1.13	–0.13	0.67	2.72	0.67	0.24	–0.58	3.46	0.65	–1.06	1.04
Games and related activities that require social interaction	4.69^a^	1.28	–0.42	–0.10	3.07^a^	0.65	–0.23	0.02	3.63^a^	0.63	–1.93	4.08^c^
Games that involve putting/adding on your body in relation to others’ bodies	3.97	1.27	–0.21	0.52	2.46	0.75	0.31	–0.22	3.20	0.82	–0.87	0.27
Guided imagining	3.58	1.16	–0.02	–0.06	2.30	0.73	0.38	0.07	2.91	0.78	–0.60	0.29
Long form improvisation games	3.07	1.15	–0.13	–0.59	2.05	0.69	0.35	0.25	2.46	0.87	–0.20	–0.71
Memorizing lines	3.88	1.39	–0.04	–0.40	2.17	0.91	0.37	–0.64	3.19	0.82	–0.84	0.20
Modeling/demonstration – audio or video resources	2.98	1.14	0.19	–0.19	1.89	0.64	0.44	0.84	2.46	0.94	–0.28	–0.93
Modeling/demonstration – peer	4.21	1.22	–0.16	0.35	2.53	0.71	0.21	–0.25	3.43	0.76	–1.43	1.99
Modeling/demonstration – teacher	4.08	1.47	0.12	–0.51	2.14	0.76	0.46	0.14	2.96	0.91	–0.72	–0.13
Performance for the class	4.62^a^	1.26	–0.46	0.50	2.93^a^	0.74	–0.12	–0.60	3.73^a^	0.58	−2.32^b^	5.76^c^
Performance for the public	3.32	1.19	0.38	0.17	2.82	0.84	–0.25	–0.55	2.98	0.90	–0.64	–0.27
Physical conditioning	3.12	1.44	0.41	–0.59	2.19	0.71	0.46	0.37	2.69	0.95	–0.39	–0.71
Reading a script, either silently or aloud	4.03	1.18	–0.32	0.17	2.37	0.73	0.70	0.23	3.26	0.81	–1.00	0.57
Reflection	5.00^a^	1.31	–0.66	0.53	3.24^a^	0.74	–0.53	–0.62	3.68^a^	0.61	−2.03^b^	4.13^c^
Rehearsing work for performance	4.53	1.08	–0.30	1.25	2.86	0.73	0.004	–0.63	3.58	0.71	–1.92	3.75^c^
Relaxation techniques and deep breathing	4.19	1.29	–0.11	–0.53	2.62	0.74	–0.16	–0.20	3.24	0.74	–0.91	0.86
Role play games	4.09	1.23	–0.53	0.18	2.70	0.69	0.21	–0.51	3.28	0.80	–1.19	1.36
Scene study	4.49	1.07	–0.07	0.94	2.72	0.74	0.39	–0.86	3.54	0.67	–1.46	2.15^c^
Script/character analysis	4.59	1.06	–0.15	0.68	2.80	0.75	0.03	–0.64	3.71^a^	0.59	−2.25^b^	5.27^c^
Sensory or memory recall	3.56	1.19	0.18	0.32	2.31	0.76	0.31	–0.12	2.91	0.88	–0.67	–0.07
Short form improvisation games	4.13	1.14	–0.39	0.42	2.38	0.70	0.39	0.06	3.23	0.76	–0.94	0.88
Speed throughs	2.68	1.12	0.23	–0.61	1.62	0.65	0.75	0.25	2.30	0.88	–0.21	–1.03
Writing/developing original material	3.64	1.25	–0.25	–0.05	2.74	0.79	0.04	–0.64	2.94	0.94	–0.66	–0.38

*^a^Highest Rated, ^b^Skewness >2, ^c^Kurtosis >2.*

**FIGURE 1:**
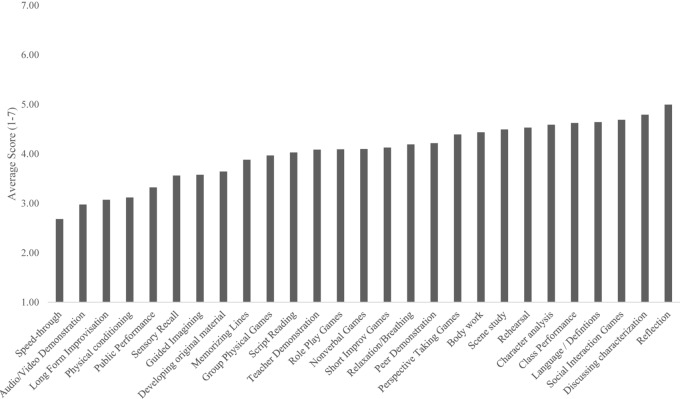
Average endorsed use of mechanistic class activities.

**FIGURE 2:**
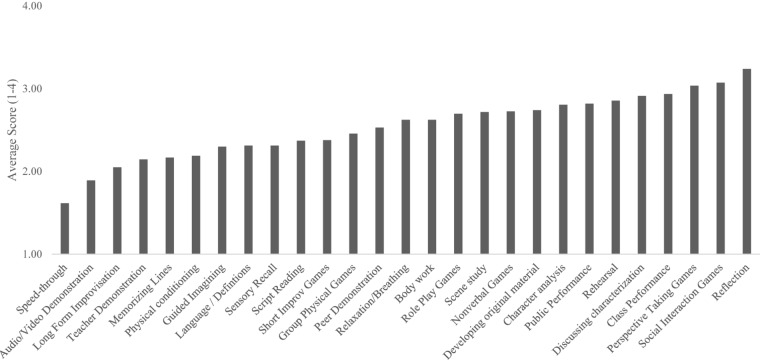
Average endorsed importance of mechanistic class activities.

**FIGURE 3:**
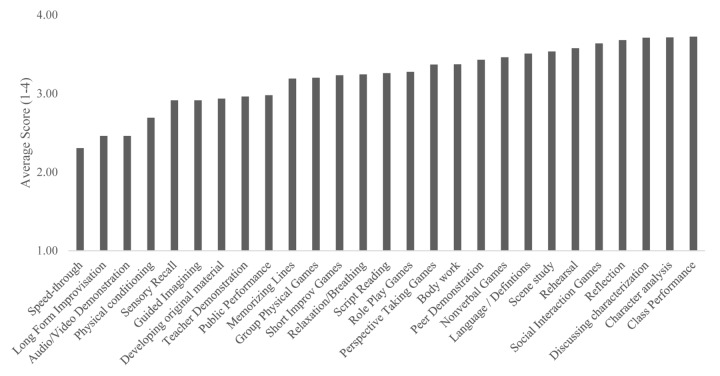
Average endorsed causal impact of mechanistic class activities.

Looking at average teacher endorsement of use (*N* = 216, see Figure 1), importance (*N* = 185, see Figure 2), and impact (*N* = 138, see Figure 3), respectively, teachers endorsed the 27 mechanistic classroom behaviors slightly differently depending on how they were asked. When using the 1–7 Likert scale for how often they used each activity (*N* = 216), teachers reported using Reflection (*M* = 5.00), Exploring/Discussing Characterization (*M* = 4.79), Social Interaction Games (*M* = 4.69), Defining Language (*M* = 4.64), Class Performance (*M* = 4.62), and Character Analysis (*M* = 4.59) most often. See Figure 1 for average endorsement scores for use of mechanisms.

When then asked which activities were most important and core to class (*N* = 185, rated 1–4), teachers rated Performance for the Class (*M* = 3.72), Exploring/Discussing Characterization (*M* = 3.71), Script/Character Analysis (*M* = 3.71), Reflection (*M* = 3.68), Social Interaction Games (*M* = 3.07), and Perspective Taking Games (*M* = 3.04) as most core to class. Please see Figure 2 for average endorsed scores for importance of all mechanistic class activities.

Finally, when asked how much each activity was instrumental in causing change (*N* = 138, rated 1–4), participants rated more than half of the activities as between “3, definitely causes change,” and “4, is THE MOST critical aspect of acting classes causing change,” with Reflection (*M* = 3.24), Social Interaction Games (*M* = 3.07), Perspective Taking Games (*M* = 3.04), In-Class Performance (*M* = 2.93), and Exploring/Discussing Characterization (*M* = 2.91) as most highly endorsed. Please see Figure 3 for average endorsed scores for the causal impact of all mechanisms.

This means, across the three ways of asking about mechanisms, teachers indicated different sets of activities depending on how they were asked but ranked almost every activity highly, well above the mean of the scale. However, a few activities were seen repeatedly as the most highly ranked—Social Interaction and Perspective Taking Games, Discussion of Characterizations, Performance in Class, and Reflection.

### Outcomes of Acting Classes

Please see Table 2 for means, standard deviations, skewness, and kurtosis for all outcome variables. Non-normality was again an issue for endorsing outcomes of theater classes. Responses for theater’s effects on Collaboration (skewness = −2.75, kurtosis = 10.26), Interpersonal Skills (skewness = −1.85, kurtosis = 4.77), Confidence (skewness = −1.62, kurtosis = 3.41), Imagination/Creativity (skewness = −1.86, kurtosis = 2.66), Emotion Recognition (skewness = −1.43, kurtosis = 2.81), Emotion Expression (skewness = −1.17, kurtosis = 2.09), Self-Esteem (skewness = −1.78, kurtosis = 4.73), Trust in Others (skewness = −1.62, kurtosis = 4.24), and Empathy (skewness = −1.82, kurtosis = 4.95) were negatively skewed and leptokurtic. In general, participants’ responses for these variables were clustered heavily around the mean. This means, again, that almost every teacher was rating all of these variables as highly likely to change as a result of theater classes, with very little variance in ratings.

**TABLE 2:** Descriptive statistics for outcome variables (in alphabetical order).

		Positively Affected				Ranking		
Variables	Mean	SD	Skewness	Kurtosis	Mean	SD	Skewness	Kurtosis
Academic performance	5.35	1.22	–0.63	0.741	18.74	6.45	–0.32	–0.70
Collaboration	6.65^a^	0.73	−2.75^b^	10.26^c^	6.98^a^	6.71	1.32	0.74
Communication skills	6.38^a^	0.86	–1.32	1.19	9.33	7.21	0.91	–0.15
Confidence	6.43^a^	0.85	–1.62	3.41^c^	8.93^a^	7.24	0.85	–0.43
Develop acting skills	6.23	1.07	–1.32	1.26	12.46	8.36	0.24	–1.16
Emotion expression	6.00	1.06	–1.17	2.09^c^	14.71	7.52	0.23	–0.92
Emotion recognition	5.84	1.24	–1.43	2.81^c^	14.99	7.40	0.16	–1.10
Emotion regulation	5.26	1.40	–0.69	0.32	17.91	7.03	–0.43	–0.79
Empathy	6.35	0.96	–1.82	4.95^c^	9.17^a^	6.72	0.72	–0.48
Expressive language	5.98	1.06	–0.97	1.10	16.02	7.49	–0.30	–1.04
Eye contact	5.85	1.25	–1.16	1.43	15.59	7.45	0.07	–1.11
Focus on task	5.78	1.11	–0.66	–0.27	16.11	6.91	–0.03	–0.95
Imagination/Creativity	6.58^a^	0.78	–1.86	2.66^c^	7.89^a^	6.94	1.07	–0.02
Imitation skills	5.28	1.33	–0.53	–0.22	19.06	7.33	–0.71	–0.55
Interpersonal skills	6.25	1.04	–1.85	4.77^c^	11.29	7.00	0.49	–0.69
Language comprehension	5.77	1.08	–0.79	1.07	17.95	6.62	–0.64	–0.22
Matching of physical body (including face) to emotional state	5.53	1.32	–0.99	1.14	17.26	7.38	–0.33	–0.96
Memory	5.53	1.36	–0.81	0.40	17.72	7.26	–0.61	–0.51
Motor skills	5.20	1.27	–0.61	0.58	20.00	6.91	–0.68	–0.69
Paying attention	5.69	1.19	–0.80	0.57	15.15	7.70	–0.06	–1.26
Physical control	5.54	1.14	–0.49	–0.06	19.53	6.52	–0.85	0.13
Resilience	5.77	1.20	–0.99	1.23	15.66	7.61	–0.13	–1.10
Self-control	5.50	1.18	–0.62	0.46	16.20	6.93	–0.25	–0.68
Self-esteem	6.17	1.08	–1.78	4.73^c^	11.92	8.03	0.38	–1.10
Self-reflection on work	6.01	1.10	–0.96	0.15	13.22	7.06	0.25	–0.76
Self-understanding	6.03	1.07	–0.96	0.40	12.03	7.51	0.39	–0.88
Trust in others	6.07	1.11	–1.62	4.24^c^	12.60	6.92	0.45	–0.75
Turn taking	5.49	1.31	–0.63	0.02	17.44	7.70	–0.26	–1.24

*^a^Highest Rated, ^b^Skewness >2, ^c^Kurtosis >2.*

**FIGURE 4:**
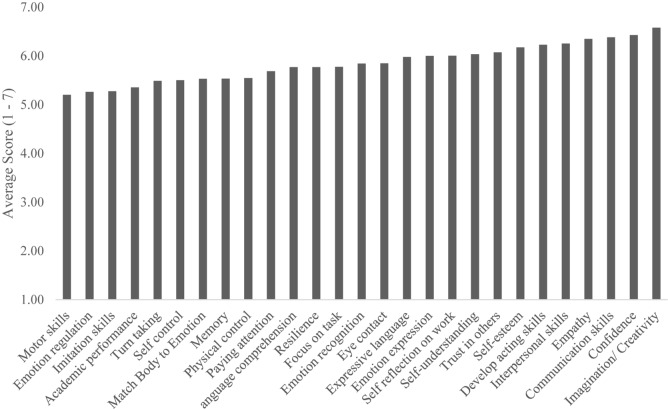
Average endorsed positive outcomes from acting classes.

Similarly to mechanism ratings, when teachers were asked to rate the 28 possible outcomes of theater classes on a 1–7 Likert-type scale (*N* = 178), they rated almost all outcomes as extremely likely to occur. The highest-endorsed outcome was Collaboration (*M* = 6.65), and the lowest was Motor Skills (*M* = 5.20), meaning that there was only a 1.45-point difference (out of a six-point possible spread) between the highest- and lowest-endorsed outcomes. Teachers simply did not use the full scale (as can be seen in our statistics of non-normality, above). When looking at the top outcomes on the Likert-type ratings, theater teachers rated Collaboration (*M* = 6.65), Imagination/Creativity (*M* = 6.58), Confidence (*M* = 6.43), Communication (*M* = 6.38), Empathy (*M* = 6.35), and Interpersonal Skills (*M* = 6.25) as most affected by theater activities and experiences. See Figure 4 for average scores for endorsement of positive impact of all outcomes.

**FIGURE 5:**
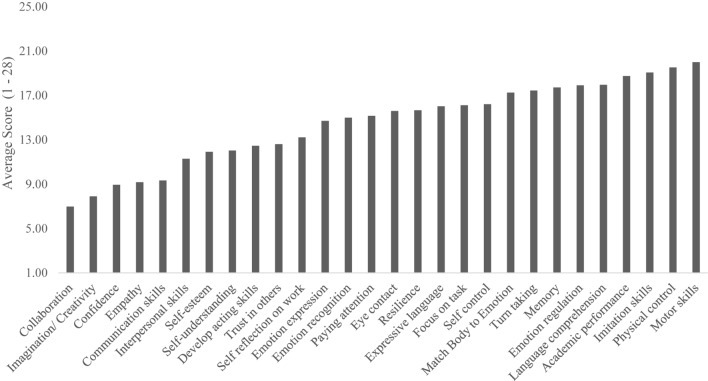
Average endorsed ranking of positive outcomes (Lower Number = Better Ranking).

We also forced teachers to rank-order all the outcomes, from 1 (most likely to change) to 28 (least likely to change), N = 148. We hoped this would enable teachers to be more fine-grained in their analyses of which outcomes change as a result of theater, rather than globally endorsing theater as positive for all outcomes (which we hypothesized they would do, and they did). When looking at which outcomes were ranked #1 most often, Imagination/Creativity (*N* = 25), Developing Acting Skills (*N* = 20), Empathy (*N* = 20), and Confidence (*N* = 15) were the top four. When looking at the average rankings (where lower is better), Collaboration (*M* = 6.98), Creativity (*M* = 7.89), Confidence (*M* = 8.93), Empathy (*M* = 9.17), and Communication (*M* = 9.33) were the top five, the same top five as the Likert-type rating. As a note, Developing Acting Skills was not as highly ranked (*M* = 12.46). See Figure 5 for average endorsed ranking scores for all outcomes.

These results again show that theater teachers strongly believe in the power of theater to cause change, with high rankings for most outcomes. However, Creativity, Confidence, Collaboration, Communication, and Empathy seem to emerge as top rated.

Interestingly, although it had been previously proposed by previous research as likely to change as a result of acting classes, Academic Performance (*M* = 18.74) was not ranked highly. Verbal Outcomes (*M* = 17.95) and Emotion Regulation (*M* = 17.91), where there is actual causal evidence of positive change (Podlozny, 2000; Goldstein and Lerner, 2017), were also not at the top of teachers’ rankings. This may be due to differences in the level and type of teaching our participants are engaged in.

### Differences by Teacher Variables

We then investigated if teacher differences in level taught (i.e., elementary, middle, and high school) were driving results. However, we were limited by the data collected. We conducted an exploratory analysis using teacher education as a predictor of time spent with different age groups. Using a smaller sample of 137 participants who provided their education information, we first conducted a one-way ANOVA to assess whether teachers with different levels of education (i.e., AA/BA, MA, and Ph.D.) spent significantly different amounts of time with certain age groups. There were overall differences between teacher education levels and percent of time teaching adolescents [*F*(2,132) = 9.92, *p* < 0.001] and professional students [*F*(2,132) = 10.38, *p* < 0.001]. To further examine differences in time teaching adolescents, we used Bonferroni *post hoc* comparisons. Comparisons revealed that teachers with AAs and BAs (*M* = 41.75%, *SD* = 44.73%) spent significantly less time with adolescents than those with MAs/MFAs (*M* = 71.60%, *SD* = 38.13%; *p* < 0.01) and Ph.D.’s (*M* = 92.50%, *SD* = 21.59%; *p* < 0.001). Also, respondents with MAs/MFAs reported spending marginally less time with adolescents than those with Ph.D’s (*p* = 0.057). When examining differences in time teaching professional students, *post hoc* comparisons revealed that those with AAs/BAs (*M* = 33.00%, *SD* = 45.92) reported spending significantly more time teaching professionally than those with MAs/MFAs (*M* = 7.47%, *SD* = 20.94; *p* < 0.001) and Ph.D’s. (*M* = 1.59%, *SD* = 4.73; *p* < 0.001). Those with MAs and Ph.D’s did not significantly differ in their reported time spent teaching professionals. This means that respondents with higher levels of education spent more time teaching in high school and college classrooms than those with lower levels of education, who were more focused on younger children, mainly in professional settings. As such, we continued to explore differences based on teacher level of education, and thus their time teaching at different levels or age groups.

#### Effects of Teacher Education

Because our analyses suggested significant differences in level of teaching between different types of teacher education, we then conducted analyses by teacher level of education, to see if teacher education is associated with differences across endorsed mechanisms and outcomes from acting classes. While there are individual differences across many of the different mechanisms and outcomes, we focus in on those ranked as the most important.

##### Mechanisms

We began with one-way ANOVAs to compare teacher ratings of mechanisms across levels of education (i.e., AA/BA, MA, and Ph.D.). For these analyses, we used a composite average across the three activity questions (normed to account for different scales).

There were significant differences in teachers’ perceptions of activity importance by level of education. One-way ANOVAs revealed significant overall differences in perceptions of importance for Exploring/Discussing Characterization [*F*(2,132) = 6.63, *p* < 0.01] and Public Performance [*F*(2,133) = 5.57, *p* < 0.01]. Bonferroni *post hoc* comparisons were used to assess mean differences between the different education levels. For Exploring/Discussing Characterization, those with MAs (*M* = 0.17, *SD* = 0.68) endorsed this mechanism significantly more than those with AA/BAs (*M* = −0.36, *SD* = 0.81; *p* < 0.05) and PhDs (*M* = −0.30, *SD* = 0.93; *p* < 0.05), who did not differ in their endorsement. For endorsement of Public Performances, those with AA/BAs (*M* = 0.52, *SD* = 0.64) endorsed this mechanism significantly more than those with MAs (*M* = 0.01, *SD* = 0.73; *p* < 0.05) or Ph.D’s (*M* = −0.21, *SD* = 0.93; *p* < 0.01), who did not differ from each other.

##### Outcomes

When asked about the outcomes being affected by theater classes, one-way ANOVA revealed significant overall differences in Communication Skills [*F*(2,134) = 5.53, *p* < 0.01], Interpersonal Skills [*F*(2,134) = 8.54, *p* < 0.001], Confidence [*F*(2,134) = 6.13, *p* < 0.01], Imagination/Creativity [*F*(2,134) = 7.23, *p* = 0.001], and Empathy [*F*(2,134) = 4.28, *p* < 0.05] by education level.

*Post hoc* comparisons revealed that those with MAs reported Empathy (M_*AA/BA*_ = 6.38, *SD* = 0.92; M_*MA*_ = 6.51, *SD* = 0.75; M_*Ph*_._*D*_. = 5.87, *SD* = 1.49) as being significantly more affected by theater classes than those with PhDs (*p* < 0.05). However, respondents with AA/BAs did not differ from those with MAs or those with Ph.D’s.

Respondents with AA/BA and MAs rated Communication Skills (M_*AA/BA*_ = 6.62, *SD* = 0.59; M_*MA*_ = 6.49, *SD* = 0.78; M_*Ph*_._*D*_. = 5.91, *SD* = 1.08; *p*_*AA/BA*_ < 0.05, *p*_*MA*_ < 0.01), Interpersonal Skills (M_*AA/BA*_ = 6.43, *SD* = 0.87; M_*MA*_ = 6.40, *SD* = 0.85; M_*Ph*_._*D*_. = 5.43, *SD* = 1.65; *p*_*AA/BA*_ < 0.01, *p*_*MA*_ < 0.001), Confidence (M_*AA/BA*_ = 6.71, *SD* = 0.64; M_*MA*_ = 6.51, *SD* = 0.72; M_*Ph*_._*D*_. = 5.91, *SD* = 1.28; *p*_*AA/BA*_ < 0.01, *p*_*MA*_ < 0.01), and Imagination/Creativity (M_*AA/BA*_ = 6.81, *SD* = 0.51; M_*MA*_ = 6.70, *SD* = 0.59; M_*Ph*_._*D*_. = 6.13, *SD* = 1.10; *p*_*AA/BA*_ < 0.01, *p*_*MA*_ < 0.01) as being significantly more affected than those with Ph.D’s. However, there were no significant differences between AA/BAs and MAs in perceived effect of theater classes in Communication Skills, Interpersonal Skills, Confidence, and Imagination/Creativity.

## Discussion

Taken together, a few patterns emerge in our study of teachers’ perceptions of acting classes. The first, and largest, finding is that teachers almost universally believe that every possible outcome from acting classes is occurring. Across the 28 possible outcomes, there was consistently high endorsement, with teachers using only the upper end of the rating scale. The lowest-endorsed outcome was still significantly above the midpoint of the scale. Teachers also believe most activities within acting classes are important. Across the 27 classroom activities that may be mechanisms for changing outcomes, there was high endorsement of their impact, with average ratings for even the lowest-endorsed mechanistic activity above the midpoint of the scale. This high endorsement was retained regardless of how we asked the question. Teachers seem to believe that all activities they engage in during an acting class are almost equally important to learning and that practically every possible outcome from acting classes is occurring in some way. This is seen in the kurtosis and skewness of our data. Yet simultaneously, there were a few emergent patterns that point toward both mechanistic activities in acting classes that could be used in future intervention work and for target outcomes for researchers interested in the effects of acting training to focus their energies.

First, the most commonly endorsed outcomes that may occur as a result of theater participation are Creativity, Confidence, Collaboration, Communication, and Empathy. Theoretically, these make sense as endorsed outcomes. Acting and theater are activities of creation, and students must be taught to believe in themselves and their creative impulses. Hence, creativity and confidence. Acting and theater are social enterprises, requiring group work and interaction as a baseline to creation. Hence, collaboration, communication, and empathy. A growing body of work has pointed to the social aspects of acting as generalizable outcomes affected by theater classes (e.g., Hui and Lau, 2006; Goldstein and Winner, 2012; Goldstein and Lerner, 2017; Rowe et al., 2018; Van de Vyver and Abrams, 2018). Critically, these findings link back to the one large-scale landscape study of theater classrooms (Omasta, 2012), which found that teachers endorsed Creativity, Collaboration, and Confidence as outcomes of theater classes. In light of these findings, acting teachers might consider specifically focusing on such social and emotional skills as intended goals of their outcomes by intentionally incorporating them into lessons. These outcome skills are all interrelated as well—the confidence to be creative and the need for empathy while engaged in collaborative activities show the ways in which acting classes can be considered holistic teaching opportunities for students to practice integrating various social and emotional skills into their daily lives.

Yet many of the areas previously highlighted as outcomes of theater participation were not endorsed by teachers as being the outcomes most likely to change. Language outcomes, including expressing and understanding language, and memory have both been extensively studied as positive outcomes from theater education and experience (e.g., Noice et al., 1999; Podlozny, 2000). While both highly endorsed (because everything was highly endorsed), neither was singled out as a particularly strong outcome of theater education. Similarly, overall academic performance was not particularly strongly endorsed as an outcome of theater, although of course, as all outcomes were highly endorsed, it was also not ignored. It may be that these commonly discussed outcomes were not rated as highly because teachers do not see immediate effects of their classes on vocabulary, academic performance, or memory in class, or because they cannot see how students do outside of their classes. However, it may also be that teachers focus their classrooms on other outcomes rather than academic ones. Researchers, policy advocates, and educators would do well to focus on the uniqueness and specificity of the effects of this art form, rather than hoping for global outcomes or outcomes that are not necessarily related to classroom behaviors and activities that are mechanisms for change. Teachers, too, may have specific perspectives about the most likely outcomes to change, while research directly with student outcomes may show findings on other outcomes, such as academic progress.

Within mechanisms, the classroom activities of Reflection, Discussing and Analyzing Characterizations, and Social Interaction and Perspective Taking Games were most often noted as mechanistically important within a classroom. Reflection involves metacognitive thinking about words, characters, actions, and processes. It is a standard educational tool used for deep thinking and higher-order understanding (Kish et al., 1997; Quinton and Smallbone, 2010; Bertucci et al., 2012; Wade-Jaimes et al., 2018). It is not surprising, therefore, that acting teachers think of Reflection as critical to their process. However, Reflection is not particularly an acting or even artistic enterprise (despite being a critical part of other studies of the habits of mind involved in art making; Hetland et al., 2007). So, while critical to outcomes in acting, Reflection is not necessarily acting itself. Similarly, Social Interaction and Perspective Taking Games are not acting *per se* but, rather, are exercises meant to prepare students for acting. These build the skills of acting and the group and ensemble requirements for a group of actors to work together, which can then be taken into a scene (Spolin, 1963/1999).

More directly related to acting and performance is the endorsement for Analyzing and Creating Characterizations as a critical mechanism. Characterization involves thinking about characters, creating the mental, emotional, and behavioral lives of the people who must then be portrayed onstage. This really is the work of acting most directly. Students must learn how to figure out a character and create a portrayal before they begin to embody it. Yet even within characterization work, there are a number of key other psychological and behavioral skills, such as using theory of mind to think about characters, understanding personality and situational constraints on behavior, and placing characters in the right time and context for the play being performed. Each of these behaviors within the task of discussing, analyzing, and creating characterization is ripe for further inquiry, investigating component psychological requirements, and how teacher and student approach the task at hand.

While the results are not as clearly delineated as scientists of education and developmental psychology might hope, they also point to a real opportunity for future research. The description of specific outcomes and mechanisms that are core to theater classrooms, at least according to teachers who run those classrooms, provides an important roadmap for future research. The field must begin to link possible mechanisms of change to possible student outcomes to move toward causal explanations of what theater does for development and how it achieves those positive effects.

### Limitations

This study was of teachers’ perceptions—what they believe is happening in their classroom that is important and what they believe is changing outside of the classroom as a result. These opinions can be biased both by teachers’ belief in the arts as a high-impact practice and by defensiveness about the importance of their programs (Holcomb, 2007; Hayford and Kattwinkel, 2018). More direct naturalistic measurement of what is actually occurring inside of classrooms and more mechanistically driven outcome research are still missing. An important follow-up to this study is pairing these findings with work that directly looks at classroom activities and student outcomes (as we are currently conducting in our research group). Such a pairing will be necessary as the field builds a psychology of acting and a full representation of the work of theater classes. Future research must also include teachers as members of the research team, rather than only as research participants. Such community–researcher partnerships (e.g., as in Olenina et al., 2019) can create a more ecologically valid research experience that illuminates important takeaways for practitioners.

An important limitation, too, is that our sample is overwhelmingly White and American, as is our research team. This means that the questions and ideas behind the survey came from a Western, text-based theater perspective, which holds at its core a focus on psychological realism. Theater outside of the United States, or taught by non-White teachers, may look fundamentally different (or be similar). Including samples and data from countries where the focus is not psychological realism may alter the results, toward self-expression or physical training. Only cross-cultural and cross-community sampling will help resolve this issue.

Our data were non-normal, and our respondents, obviously very enthusiastic about their topic. Researchers must understand stakeholder perspectives to build a research program that is sensitive and responsive to their needs and beliefs. Yet simultaneously, there is real validity to teachers’ beliefs that most activities within classes are important and most outcomes are possible. Theater in schools has long been under threat (Parsad and Spiegelman, 2012; Sparks et al., 2015) and is often cut from curricula before other art forms or integrated into other subjects (such as Science or English; Dawson, 2018) rather than taught as its own, worthy subject. Therefore, teachers are understandably defensive about their programs and have a vested interest in their success.

To overcome issues of skewed data in the future, teacher surveys could ask teachers to specifically link mechanisms to 1 of the 28 outcomes, rather than asking about each mechanism impacting the outcomes generally, or follow up on surveys with additional requests of examples of mechanisms and their direct uses in the classrooms. Future work should also try to overcome skewness by asking about outcomes which may be completely unrelated to theater (e.g., getting better at Calculus) in order to get teachers thinking about the variety of domains where theater may or may not apply. This is where a naturalistic observation methodology would also be helpful. For example, it could be that the specifics of discussions teachers are having about characterizations or within reflection activities are where collaboration, imagination, and empathy are being highlighted.

## Conclusion

This study took its inspiration from Studio Thinking (Hetland et al., 2007), a framework for arts-based inquiry which sought to discover how arts classrooms are structured and what is taught in those classrooms. This framework for inquiry resulted in an understanding of how students are taught to think like artists, how teachers organize their time and space, and the interactions that occur in visual arts classrooms. We extended this framework to theater by taking the first step of surveying teachers about the perceived outcomes of participating in theater and activities within theater classes, or mechanisms that might be responsible for those outcomes. We hope this study provides a first look into what researchers should focus on when looking for positive effects of acting classes and what kinds of behaviors and activities, drawn from acting, may be best used in interventions seeking to improve such outcomes.

## Data Availability Statement

The datasets generated for this study are available on request to the corresponding author.

## Ethics Statement

The studies involving human participants were reviewed and approved by the George Mason University IRB. The patients/participants provided their written informed consent to participate in this study.

## Author Contributions

TG developed the study. TG and BT created and distributed the survey. TG and DY conducted the data analyses. TG, DY, and BT wrote the manuscript.

## Conflict of Interest

The authors declare that the research was conducted in the absence of any commercial or financial relationships that could be construed as a potential conflict of interest.
