# Supplementary material for: It’s All Critical: Acting Teachers’ Beliefs About Theater Classes
Source: Front Psychol. 2020 May 19;11:775. doi: 10.3389/fpsyg.2020.00775 (PMC7248218; doi:10.3389/fpsyg.2020.00775)
Supplement: Supplementary file 1 [file Data_Sheet_1.docx]

**Appendix A – Full Survey**

**Mechanisms**

*Instructions: For the questions that follow, we are interested in the techniques and strategies you use in acting classes. If you teach at multiple levels, or different types of theatre/ acting classes, please answer for the class you consider to be the most advanced acting class.*

The following answer options were presented below each question:

- Not at all
- Almost Never
- Few Classes
- Some Classes
- Most Classes
- Every Class
- Multiple Times Per Class

1. How often do you use sensory or memory recall activities in your acting classes?
2. How often do you use writing/developing of original material in your acting classes?
3. How often do you use script/character analysis in your acting classes?
4. How often do you use reflection in your acting classes?
5. How often do you use games and related activities that require social interaction (e.g. ice breakers, name games) in your acting classes?
6. How often do you use games and activities that involve nonverbal skills (e.g. pantomime, speaking gibberish) in your acting classes?
7. How often do you use games and activities involving taking the perspective of other people in your acting classes?
8. How often do you use modeling/demonstration by the teacher in your acting classes?
9. How often do you use modeling/demonstration by a peer in your acting classes?
10. How often do you use modeling/demonstration from audio or video resources in your acting classes?
11. How often do you use relaxation techniques and deep breathing in your acting classes?
12. How often do you use games that involve role play in your acting classes?
13. How often do you use short form improvisation games in your acting classes?
14. How often do you use long form improvisation games in your acting classes?
15. How often do you use scene study (e.g. discussing/analyzing performances) in your acting classes?
16. How often do you use body work (e.g. instructed physical movement) in your acting classes?
17. How often do you use physical conditioning (e.g. pushups, aerobics) in your acting classes?
18. How often do you use games that involve putting/adding on your body in relation to others' bodies (e.g. building a group tableaux; machines) in your acting classes?
19. How often do you define the language of acting, or define the language of a play/performance in your acting classes?
20. How often do you engage in memorizing lines in your acting classes?
21. How often do you engage in guided imagining in your acting classes?
22. How often do you engage in speed throughs (e.g. reciting lines without characterization) in your acting classes?
23. How often do you engage in exploring/discussing characterization in your acting classes?
24. How often do you engage in rehearsing work for performance (in class or for public) in your acting classes?
25. How often do you engage in performance for the public in your acting classes?
26. How often do you engage in performance for the class in your acting classes?
27. How often do you engage in reading a script, either silently or aloud in your acting classes?

**Importance of Strategies and Mechanisms**

*How important are the following activities to your acting classes?*

Answer options were presented in a grid, from left to right as follows:

| Core to class | Incidental to class | Unimportant | Do not use |
| --- | --- | --- | --- |

Activities:

1. Sensory or memory recall activities
2. Writing/developing of original material
3. Script/character analysis
4. Reflection
5. Games and related activities that require social interaction
6. Games and activities that require nonverbal skills
7. Games that involve taking the perspective of other people
8. Modeling/demonstration by the teacher
9. Modeling/demonstration by peers
10. Modeling/demonstration by audio/video resources
11. Relaxation techniques and deep breathing
12. Games that involve role play
13. Short improvisation games
14. Long form improvisation games
15. Scene study
16. Body work
17. Physical conditioning
18. Games that involve putting/adding on one’s body in relation to others’ bodies
19. Define the language of acting, or the language of play/performance
20. Memorizing lines
21. Guided imagining
22. Speed through
23. Exploring/discussing characterization
24. Rehearsing work for performance
25. Performance for the public
26. Performance for the class
27. Reading a script, silently or aloud

**Outcomes**

*Instructions: For the questions that follow, you will be asked about what outcomes you think occur as a result of participating in acting classes. These outcomes could be for skills and abilities that occur within the acting classes, or outside of the acting classes.*

The following answer options were presented below each question:

1 – Not at all

2

3

4

5

6

7 – A great deal

1. How much do you think "eye contact" is positively affected by being in theatre classes?
2. How much do you think "focus on a task" are positively affected by being in theatre classes?
3. How much do you think "self reflection on work" are positively affected by being in theatre classes?
4. How much do you think "paying attention" are positively affected by being in theatre classes?
5. How much do you think "communication skills" are positively affected by being in theatre classes?
6. How much do you think "collaboration" are positively affected by being in theatre classes?
7. How much do you think "interpersonal skills" are positively affected by being in theatre classes?
8. How much do you think "resilience" is positively affected by being in theatre classes?
9. How much do you think "confidence" is positively affected by being in theatre classes?
10. How much do you think "imagination and creativity" are positively affected by being in theatre classes?
11. How much do you think "language comprehension" is positively affected by being in theatre classes?
12. How much do you think "expressive language" is positively affected by being in theatre classes?
13. How much do you think "memory" is positively affected by being in theatre classes?
14. How much do you think "turn taking" is positively affected by being in theatre classes?
15. How much do you think "emotion recognition" is positively affected by being in theatre classes?
16. How much do you think "emotion expression" is positively affected by being in theatre classes?
17. How much do you think "emotion regulation" are positively affected by being in theatre classes?
18. How much do you think "imitation skills" are positively affected by being in theatre classes?
19. How much do you think "self-esteem" is positively affected by being in theatre classes?
20. How much do you think "self-understanding" are positively affected by being in theatre classes?
21. How much do you think "motor skills" are positively affected by being in theatre classes?
22. How much do you think "physical control" is positively affected by being in theatre classes?
23. How much do you think "matching of physical body (including face) to emotional state" is positively affected by being in theatre classes?
24. How much do you think "trust in others" are positively affected by being in theatre classes?
25. How much do you think "academic performance" is positively affected by being in theatre classes?
26. How much do you think "acting skills" are positively affected by being in theatre classes?
27. How much do you think "empathy" is positively affected by being in theatre classes?
28. How much do you think "self-control" is positively affected by being in theatre classes?

*Instructions: Please rank order all of the above mentioned outcomes from theatre classes, from MOST (#1) likely to occur, to LEAST (#28) likely to occur. Drag and drop each outcome to its desired location.*

______ Eye contact

______ Paying attention

______ Communication skills

______ Collaboration

______ Interpersonal skills

______ Resilience

______ Confidence

______ Imagination/ Creativity

______ Language comprehension

______ Expressive language

______ Memory

______ Turn taking

______ Emotion recognition

______ Emotion regulation

______ Imitation skills

______ Self-esteem

______ Self-understanding

______ Motor skills

______ Matching of physical body (including face) to emotional state

______ Trust in others

______ Academic performance

______ Develop acting skills

______ Empathy

______ Self control

______ Self reflection on work

______ Focus on task

______ Emotion expression

______ Physical control

**Mechanisms and Outcomes**

*Instructions: Thinking about the outcomes mentioned above, how much do you think each of the activities you answered about are instrumental or causal in creating change?*

| Does not cause change | Sometimes causes change | Definitely Causes Change | Is THE MOST critical aspect of acting classes causing change |
| --- | --- | --- | --- |

Answer options were presented in a grid, from left to right as follows:

Activities:

1. Sensory or memory recall activities
2. Writing/developing of original material
3. Script/character analysis
4. Reflection
5. Games and related activities that require social interaction
6. Games and activities that require nonverbal skills
7. Games that involve taking the perspective of other people
8. Modeling/demonstration by the teacher
9. Modeling/demonstration by peers
10. Modeling/demonstration by audio/video resources
11. Relaxation techniques and deep breathing
12. Games that involve role play
13. Short improvisation games
14. Long form improvisation games
15. Scene study
16. Body work
17. Physical conditioning
18. Games that involve putting/adding on one’s body in relation to others’ bodies
19. Define the language of acting, or the language of play/performance
20. Memorizing lines
21. Guided imagining
22. Speed through
23. Exploring/discussing characterization
24. Rehearsing work for performance (in class or for public)
25. Performance for the public
26. Performance for the class
27. Reading a script, silently or aloud

Open-Ended Question: If you'd like to specify which kinds of activities are causing which kinds of change, please feel free to tell us as much or as little as you'd like below.

________________________________________________________________

**Demographics**

Please enter your gender

- Male
- Female
- Other
- Prefer not to answer

Please enter your age

________________________________________________________________

Please pick as many categories as you feel best describes your race and/or ethnicity

- Black, Carribean, or African American
- White or European American
- East Asian, Chinese, Japanese, Korean
- Southeast Asian, Indian, Pakistani, Bangladeshi
- Hispanic
- Native American
- Pacific Islander or Native Hawaiian

Please tell us how you identify your race or ethnicity

________________________________________________________________

Please choose which type(s) of schools you work in. (Pick as many as are applicable)

- Elementary
- Middle School
- High School
- College/University
- Professional Conservatory Classes for Adults
- Professional Theatre Classes for Children (e.g., Acting classes as a professional theatre)
- Community Center Theatre Classes (e.g., YMCA)

Please describe how you split your time among the multiple options above, if applicable  (e.g. 50% Middle School, 20% Professional Theatre Classes for Children, 30% Community Center Theatre Classes)

Do you have a degree in acting or performance?

- Yes
- No

Do you have a degree in directing?

- Yes
- No

Do you have a degree in theatre education?

- Yes
- No

Do you have a degree in theatre other than acting, directing, or theatre education?

- Yes (please describe) ___________________
- No

Do you have a degree in general education or some other type of education (e.g. English, Science, Elementary etc)

- Yes
- No

Please select your highest level of education

- High School Diploma
- AA
- BA, BS, BFA
- MA, MS, MFA
- PHD, JD, MBA, MD

What types of classes do you teach? Please enter the percentage of time you spend on each type of class, to equal 100% of your teaching time.

- Script based performance classes : _______
- Improvisation Classes : _______
- Movement classes : _______
- Voice Classes : _______
- Other : _______
- Total : ________

What country do you teach in?

- USA
- Canada
- Australia
- Republic of Ireland
- United Kingdom
- Other (Please specify) ___________________________
